# Psoriatic Arthritis and Metabolic Syndrome: Is There a Role for Disease Modifying Anti-Rheumatic Drugs?

**DOI:** 10.3389/fmed.2021.735150

**Published:** 2021-08-30

**Authors:** Fabiola Atzeni, Elisabetta Gerratana, Ignazio Francesco Masala, Sara Bongiovanni, Piercarlo Sarzi-Puttini, Javier Rodríguez-Carrio

**Affiliations:** ^1^Rheumatology Unit, Department of Experimental and Internal Medicine, University of Messina, Messina, Italy; ^2^Trauma and Orthopedic Unit, Santissima Trinità Hospital, Cagliari, Italy; ^3^Rheumatology Unit, Azienda Socio Sanitaria Territoriale (ASST)-Fatebenefratelli L. Sacco University Hospital, University of Milan, Milan, Italy; ^4^Department of Functional Biology, Immunology Area, Faculty of Medicine, University of Oviedo, Oviedo, Spain; ^5^Area of Metabolism, Instituto de Investigación Sanitaria del Principado de Asturias (ISPA), Oviedo, Spain

**Keywords:** metabolic syndrome, psoriatic arthritis, dyslipidemia, diabetes, hypertension

## Abstract

Although psoriatic arthritis (PsA) primarily leads to joint and skin damage, it is associated with higher prevalence of metabolic syndrome (MetS) and its components, namely hypertension, dyslipidemia, obesity, and type II diabetes. Additionally, chronic inflammation is known to aggravate these cardiometabolic factors, thus explaining the enhanced cardiovascular (CV) morbidity and mortality in RA. Furthermore, emerging evidence suggest that some risk factors can fuel inflammation, thus pointing to a bidirectional crosstalk between inflammation and cardiometabolic factors. Therefore, dampening inflammation by disease-modifying anti-rheumatic drugs (DMARDs) may be thought to ameliorate MetS burden and thus, CV risk and disease severity. In fact, recommendations for PsA management emphasize the need of considering comorbidities to guide the treatment decision process. However, the existing evidence on the impact of approved DMARDs in PsA on MetS and MetS components is far from being optimal, thus representing a major challenge for the clinical setting. Although a beneficial effect of some DMARDs such as methotrexate, TNF inhibitors and some small molecules is clear, no head-to-head studies are published and no evidence is available for other therapeutic approaches such as IL-23 or IL-17 inhibitors. This narrative review summarizes the main evidence related to the effect of DMARDs on MetS outcomes in PsA patients and identify the main limitations, research needs and future perspectives in this scenario.

## Introduction

Psoriatic arthritis (PsA) is a chronic inflammatory musculoskeletal and skin disease, associated with psoriasis (PsO). PsA can affect peripheral joints, entheses, and the axial skeleton, and it is characterized by different clinical manifestations and a variable clinical course (1). It affects 10–40% of PsO patients, although in some cases it may occur in the absence of skin manifestations. In most cases, skin manifestations precede arthritis, in 15% of the cases the onset is simultaneous, and in 10–15% of the cases arthritis precedes PsO (2). Moreover, beyond musculoskeletal and skin manifestations, patients of PsA have a higher prevalence of comorbidities compared to the general population (3), with more than half of PsA patients reporting at least one comorbidity and up to 40% of patients having more than three comorbidities (4). PsA patients exhibit a high prevalence of cardiovascular (CV) risk factors, including the metabolic syndrome (MetS) (5).

## Metabolic Syndrome

MetS is defined as a constellation of interrelated alterations, which directly increase the risk of CV disease and type II diabetes mellitus (DM) (6). The main components of MetS are: atherogenic dyslipidemia (hypertriglyceridemia, increased Apo-B, low HDLc levels), altered glucose homeostasis, arterial hypertension, and chronic pro-thrombotic and pro-inflammatory state (7, 8). These are all risk factors for developing CV disease, occurring in association with each other more often than expected by chance (6). Although definitions may vary, nowadays the most widely used MetS definition was formulated by the US National Heart, Lung, and Blood Institute (NHLBI) together with the American Heart Association (AHA) (7). In accordance with the NHLBI/AHA criteria, MetS is diagnosed when three or more of the following five criteria are present:

1. Fasting blood glucose level ≥100 mg/dl or pharmacological therapy for hyperglycemia.
2. Blood pressure ≥130/86 mmHg or pharmacological therapy for hypertension.
3. Triglycerides ≥150 mg/dl or pharmacological therapy for hypertriglyceridemia.
4. High-density lipoprotein cholesterol (HDLc) <40 mg/dl in men or <50 mg/dl in women.
5. Waist circumference ≥102 cm in men and ≥88 cm in women.

Interestingly, both MetS and its components are significantly over-represented in patients with PsA than in the general population and also compared to PsO and RA patients (9–12). Approximately 24–58% of patients with PsA have MetS, whereas it is only observed in 15–24% of individuals from the general population (5, 11, 13, 14). According to a study conducted in China, the odds ratio of MetS in PsA is 2.68 (95% CI: 1.60–4.50) when compared with the general population (5). A similar picture was observed for MetS prevalence in PsA compared to RA or ankylosing spondylitis populations in an outpatient arthritis clinic cohort [odds ratio (OR): 2.44, 95% confidence interval (CI): 1.48–4.01] (4). Importantly, the MetS components seem to precede the occurrence of PsA by at least 5 years (15), and hyperlipidemia and obesity have been described as risk factors for PsA development (16).

The elevated MetS may account for the elevated CV risk observed in PsA. In fact, patients with PsA have a 55% higher probability of developing CV diseases such as ischemic heart disease, cerebrovascular events or congestive heart failure (17). Moreover, a recent meta-analysis found that PsA patients exhibit increased mortality [relative risk (RR): 1.74, 95% CI: 1.32–2.30], particularly arising from CV disease (RR: 1.84, 95% CI: 1.11–3.06) (18).

The reasons for this increased prevalence of MetS in patients with PsA is an interesting field of research. Actually, recent studies have also associated MetS components with subclinical CV outcomes (19), thus suggesting that attenuating MetS components may lead to a certain degree of CV protection.

### Diabetes

The evaluation of the individual components of MetS in PsA patients revealed that the prevalence of DM, as well as the presence of insulin resistance, is higher than in the general population (4). In addition, studies conducted on PsA patients without DM diagnosis at the time of enrollment, have shown that patients with PsA have a greater risk [hazard ratio (HR): 1.4–1.5] of developing type II DM over time compared to the general population (10, 20, 21). Moreover, this risk appears increased in women and in those with higher disease activity (20, 22). In addition to obesity and lifestyle factors, the inflammatory process related to arthritis pathogenesis may also play a key role in the risk of developing type II DM (23, 24). In 2013, a meta-analysis showed that the risk of type II DM was higher in patients with PsA (OR: 2.18; 95% CI: 1.36–3.50) compared to those with PsO (OR: 1.76, 95% CI: 1.59–1.96) (24). In a study using data from the “Consortium of Rheumatology Researchers of North America (CORRONA registry)” the prevalence of type II DM in PsA patients was also found to be higher than in those diagnosed with RA (15 vs. 11%; OR: 1.56, 95% CI: 1.07–2.28) (11).

Of note, the risk of type II DM seems to be related to disease severity in PsA, being positively associated with joint involvement and erythrocyte sedimentation rate (20, 22). Moreover, inflammation seems to trigger insulin resistance (23, 24) in this condition, hence pointing to a potential connection of MetS and inflammatory burden in PsA.

### Hypertension

Arterial hypertension (HTN) is another CV risk factor with a higher prevalence in patients with PsO and PsA compared to the general population (4, 25). Data from a large Middle-Eastern PsA cohort reported an increase in the prevalence HTN (OR: 1.51; 95% CI: 1.40–1.6), in addition to that of hyperlipidemia (OR: 1.54; 95% CI: 1.43–1.67), type II DM (OR: 1.48, 95% CI: 1.36–1.61), and obesity (OR: 1.71, 95% CI: 1.58–1.84) (26). In a Spanish monocentric study, the prevalence of HTN was found to be higher in PsA compared to PsO (29 vs. 18%, OR: 1.7, 95% CI: 1.25–2.50) (26). A higher prevalence of HTN in patients with PsA than in patients with PsO was also observed in a cohort study of the University of Toronto (OR: 2.17, 95% CI: 1.22–3.83), after adjusting the data for demographic factors, comorbidity, and pharmacological treatments (9).

In a study obtained using data from the MarketScan claims database, a higher HTN prevalence (19.9 vs. 18.6%) and incidence (79.8 vs. 74.0 per 1,000 patient-years) were observed in PsA when compared to RA (27). Analyzing the prevalence of HTN in patients with PsO, Duan and coworkers observed an elevated prevalence of HTN compared to the general population only in patients with severe psoriatic disease (OR: 1.13, 95% CI: 1.03–1.25), but not in their mild disease counterparts (OR: 1.09, 95% CI: 0.98–1.22), suggesting that a relationship between HTN and the systemic inflammatory response is also likely (28).

### Dyslipidemia

Dyslipidemia is defined as a disorder of lipid metabolism characterized by increased LDL-cholesterol (LDLc) and triglycerides levels, usually associated with decreased HDLc levels.

A higher prevalence of dyslipidemia was observed in PsA compared to both the general population (5, 10, 25, 29). A study from the MarketScan database showed a higher incidence of hyperlipidemia in PsA patients than in controls [incidence risk ratio (IRR): 1.10, 95% CI: 1.04–1.17] (27). A similar picture was observed when compared with PsO patients (4, 26, 30). In fact, dyslipidemia seems to be more prominent in PsA patients with active disease, suggesting a potential link between inflammation and lipid profiles [reviewed in (18)]. However, the study of lipids is challenging in this scenario, since inflammation can lower serum LDLc levels (31, 32), as observed in RA (33). Therefore, hypercholesterolemia as a risk for MetS and CV disease may not apply in systemic diseases and should be interpreted with caution. Moreover, beyond lipoprotein levels, PsA patients exhibit qualitative alterations in their lipid profiles, namely a HDL3 sub-fraction reduction and an increase in most dense LDL sub-fraction, these features being associated with an enhanced atherogenicity activity (34). Furthermore, numerically higher lipoprotein A [Lp(a)] serum levels have been reported in PsA (34). Moreover, dyslipidemia in PsA patients is associated with increased markers of inflammation, such as C-reactive protein (CRP), and with a higher risk of subclinical atherosclerosis (35–37).

### Obesity

Obesity is defined as a body mass index (BMI) ≥ 30 kg/m^2^. Several studies have reported a higher prevalence of obesity in patients with PsA compared to the general population (10, 11, 25, 27, 38, 39), but also compared to patients with PsO (22.68 vs. 16.75%) in a large cohort study from the UK THIN database (40). However, also patients with PsO have a higher incidence of obesity when compared to the general population (OR: 1.66, 95% CI: 1.46–1.89), as demonstrated in a systematic review with pooled data of more than 200,000 PsO patients (41). The prevalence of obesity is also higher in PsA patients than in their RA counterparts (11), as well as in other chronic inflammatory diseases (29). Obesity has been also associated with poor treatments outcomes and decreased rates of remission attainment in PsA patients undergoing TNF inhibitors (42), thus affecting not only the metabolic dimension of the disease but the inflammatory process itself.

Obesity appears to be a significant risk factor for both the development of PsA and PsO, and this risk seems to be weight-dependent, as the risk of developing PsA increases with increasing BMI (43–45). In fact, a British study reported a growing risk RR of developing PsA with increasing BMI: the RR of PsA was 1.09 (0.93–1.28), 1.22 (1.02–1.47), and 1.48 (1.20–1.81) with BMI of 25–29.9, 30.0–34.9, and 35.0 kg/m^2^ (44), respectively. Moreover, the risk of PsA in obese patients seems to decrease if the patient undergoes weight loss, as demonstrated by several studies (46, 47). The results of two broad population-based cohort studies also showed a protective effect of bariatric surgery for the development of PsA (HR 0.52, 95% CI 0.33–0.81) (46–48).

## MetS and Inflammation

**Figure 1:**
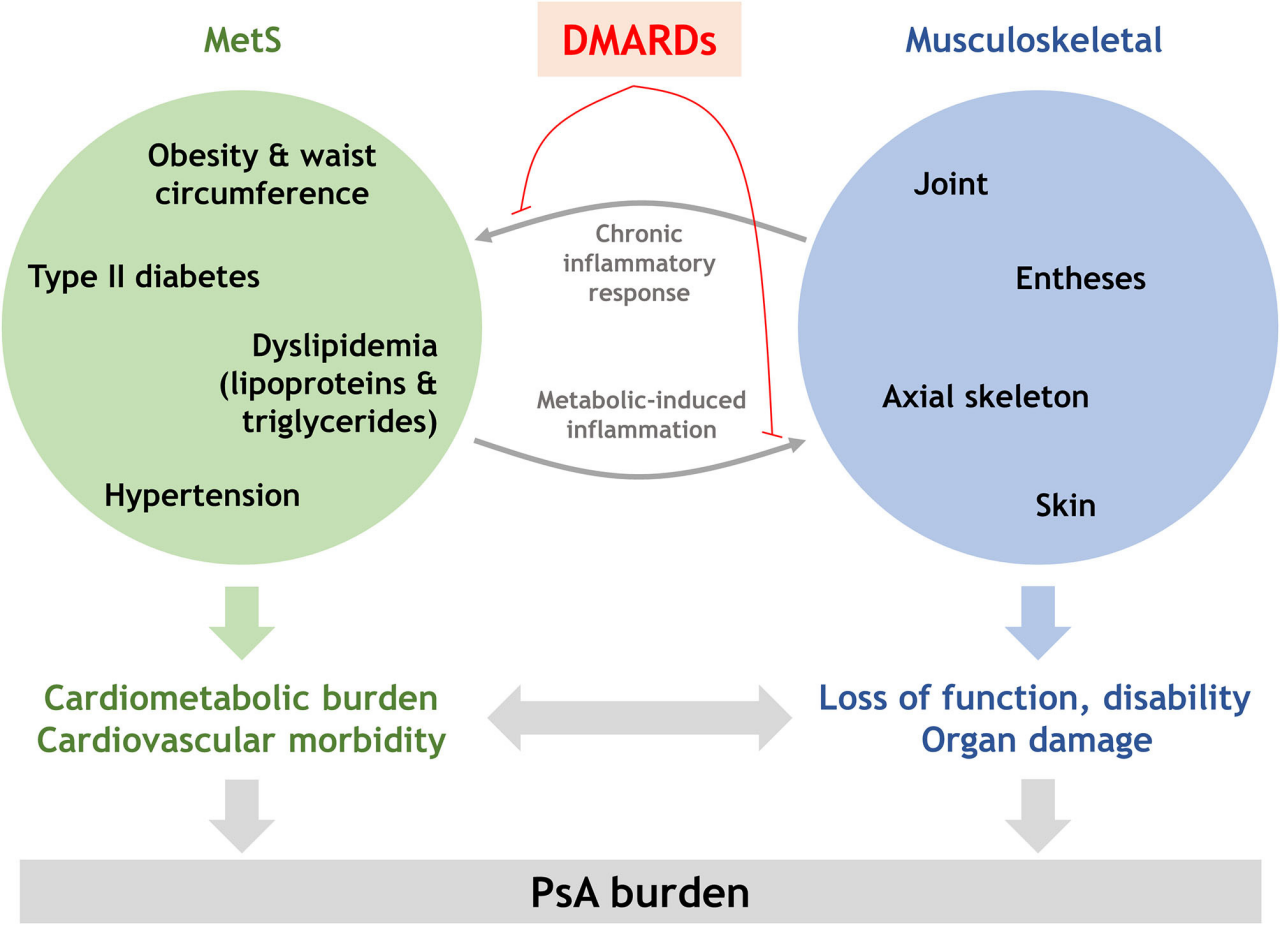
Integrative model for the interplay between musculoskeletal and MetS domains in PsA. Musculoskeletal and MetS involvement may be connected through shared inflammatory mechanisms, thus explaining the higher disease activity indices and poor therapeutic outcomes in PsA patients with MetS and the MetS aggravation in patients with active disease or higher severity. Since this crosstalk seems to be orchestrated by inflammatory circuits, dampening inflammation by DMARDs exposure may lead to a better disease control, MetS mitigation and thus, decreased PsA burden.

In addition of an enhanced CV risk, PsA patients with MetS have been reported to exhibit higher disease activity scores. In fact, there is evidence supporting that MetS occurrence is associated with PsA severity (49, 50). The underlying reasons are unclear, but several explanations might (co)exist (Figure 1). First, the composite indices to measure PsA activity contain patient reported outcomes (PROs). Obesity may contribute to the joint discomfort referred by patients, thus causing an overestimation of PROs. In addition, obesity, especially visceral obesity (51), is associated with increased CRP. Furthermore, obesity has also been associated with a lower probability of achieving a therapeutic response (52), thus accounting for an enhanced pro-inflammatory milieu as well. The increase in obesity-related PROs and CRP levels may therefore result in a higher score of the composite indices to measure disease activity (53, 54). Of note, the association between MetS and inflammation can be regarded as bi-directional, since elevated inflammation or disease severity has been associated with higher odds of DM occurrence (HR: 1.21, 95% CI: 1.03–1.41; and HR: 1.53, 95% CI: 1.08–2.18, respectively) in a recent cohort study (20), and inflammation is known to trigger impaired glycemic and lipid metabolism, hence contributing to MetS severity.

Of note, although MetS is known to predict CV disease development or subclinical atherosclerosis occurrence in PsA, inflammation has been demonstrated to play a role in shaping this association, probably by accelerating atherosclerosis development or by aggravating MetS burden (55–58). Of note, CV disease prevalence cannot be fully explained by traditional risk factors in RA (39), hence suggesting that other factors such as inflammation may play a significant role.

Mechanistic evidence adds to these findings, since a number of cytokines implicated in the psoriatic and arthritic disease domains can contribute to atherosclerosis and metabolic impairments, including Th1 (TNF, IFNγ, and IL-12) and Th17 cytokines (IL-17, IL-22, IL-23, IL-6, and TNF). These cytokines can act on distant organs, such as liver, skeletal muscle, vascular endothelia, and adipose tissue, thus bridging chronic inflammation, atherogenesis, and metabolic dysfunction leading to CV risk [reviewed in (59)].

Taken together, it is tempting to speculate that a good control of PsA may lead to an amelioration of the underlying inflammatory process thus causing an improvement of the articular and skin outcomes, but also to the MetS burden. In fact, an improvement of MetS components has been associated with reaching the minimal disease activity status in PsA (52, 60, 61), thus consequently suggesting a link between inflammation and reduction of the CV risk (62). Importantly, PsA patients not taking disease modifying anti-rheumatic drugs (DMARDs) were twice as likely to have MetS compared to PsO patients even after adjusting by age, CRP and blood pressure (adjusted OR: 2.09, 95% CI: 0.78–5.59) (19). Similarly, patients not taking DMARDs are more likely to suffer a major adverse CV event (MACE) compared to the general population (HR: 1.24, 95% CI: 1.03–1.49) after adjusting for confounders, and exhibit a numerically higher risk when compared to their DMARD-treated counterparts (HR: 1.08, 95% CI: 1.02–1.15) (40). Furthermore, a recent meta-analysis concluded that systemic therapies, including TNF inhibitors and methotrexate, was associated with a reduction of CV events, although evidence was lower than for RA patients (63). Finally, many studies linked the suppression of inflammation with a favorable impact on subclinical CV surrogate markers (18, 26, 43, 45). Taken together, all these lines of evidence support that DMARDs exposure may ameliorate MetS burden, and thus CV risk, in PsA patients (Figure 1).

Therefore, the possible presence of MetS should be seriously taken into account during the therapeutic decision process for PsA. Targeting inflammation with DMARDs may have an important effect in mitigating MetS burden in PsA.

## DMARDs and MetS in PsA

### Conventional DMARDs

#### Methotrexate

Numerous data exists supporting the role of methotrexate (MTX) in reducing CV risk in patients with chronic inflammatory diseases. The protective effect of MTX is linked to an overall reduction of the inflammatory response. Although most of the studies are derived from RA populations (64), the first data begins to appear also for patients with PsA and associated MetS.

A study assessing the safety of MTX on glucose metabolism in PsA and MetS patients found that glycated hemoglobin in such patients showed no difference before and after 12 weeks of starting treatment. As a result, the use of MTX in this category of patients is safe, having shown no hyperglycemic effects (65). It actually seems to even have a positive effect on carbohydrate metabolism. In fact, a study observed reduction of glycated hemoglobin after starting MTX treatment in patients with arthritis of about half (~0.4 units) of what would be obtained with metformin (~0.8 units). A comparable result was observed after starting treatment with TNF inhibitors (TNFi) (65). The PSARA (Psoriatic Arthritis, Ankylosing Spondylitis, Rheumatoid Arthritis Study) study, aimed at observing the effects on endothelial function of MTX in monotherapy, MTX in combination with TNFi, and TNFi in monotherapy, noted an improvement in endothelial function after 6 months of treatment in all the three treatment groups. However, this improvement was stronger in the group of patients treated with MTX in monotherapy (66).

### Biological DMARDs

#### TNF Inhibitors

The effectiveness of TNFi in patients with MetS is still a subject of debate. Several studies highlight the reduced efficacy of TNFi in obese patients (67, 68). A meta-analysis including 22 studies (for a total of 11,873 patients) conducted by Singh and coworkers showed that obesity was associated with a lower therapeutic response in patients with PsO and PsA (OR: 1.57, 95% CI: 1.30–1.89) (67). A recent study based on the US CORRONA PsA/SpA registry found that the presence of obesity was a strong predictor of failure to achieve remission in PsA (OR: 0.51, 95% CI: 0.32–0.81) (68). By contrast, an Italian study observed that the presence of MetS does not reduce the anti-inflammatory effect of TNFi neither the likelihood of reaching MDA (69). In a separate study, the same study group found out that in patients with MetS and PsA, the carotid intima-media thickness (cIMT) was greater in those treated with other DMARDs than in those undergoing TNFi, thus suggesting that the latter, by reducing inflammation may reduce CV risk in PsA (70). Whether a stronger, general effect on inflammation or a specific role of the TNF pathway was responsible of these findings remain to be elucidated. The efficacy of TNF blockade therapy in reducing or containing subclinical atherosclerosis was confirmed by other studies (71).

However, it is not yet clear whether weight-dependent changes in the dosage of the drug, possible with intravenous golimumab and infliximab, can improve the therapeutic response to TNFi in obese patients (4). Although the efficacy of TNFi may be lower in obese patients than in their non-obese counterparts, some studies have shown a lower risk of developing DM in TNFi-treated patients compared to other non-biological systemic treatments (excluding MTX) (72–74).

Interestingly, the impact of TNFi seems to be associated with a beneficial effect on several MetS components, including, including waist circumference, levels of triglycerides and HDLc as well as blood glucose levels (75). In fact, a clinical trial including 127 patients with PsA and active PsO undergoing TNFi treatment reported an increment in apolipoprotein AI, apolipoprotein B and triglycerides and a decline of Lp(a) after 12 weeks, although the relevance of these findings in terms of CV risk remained unclear (76).

#### Other Biological Drugs: IL-17 and IL-12/23 Inhibitors

Unfortunately, there is a lack of robust clinical evidence on the role of drugs targeting IL-17 and IL-12/23 on MetS and CV outcomes in PsA. Interestingly, this axis is expected to contribute to the cardiometabolic alterations, at least in PsO (77). A recent prospective study has demonstrated that overweight and obese patients had a better Disease Activity in Psoriatic Arthritis (DAPSA) score compared with their normo-weight counterparts (78), and serum IL-17 seem to correlate BMI, pointing to an association between obesity and IL-17 and thus, a potential better clinical benefit in patients with obesity. This finding was also supported by the fact that obesity was related to a Th17 expansion in adipose and peripheral tissues. However, due to the paradoxical association between IL-17 and CV disease (79, 80), whether IL-17 blockade leads to a more favorable profile and MetS mitigation requires further research.

Concerning IL-12/23 inhibitors, short-term data revealed no increased CV risk in PsO patients (81, 82). Moreover, it has been hypothesized that IL-23 inhibition may be more effective in patients with comorbidities in PsO patients (83). However, a recent *post-hoc* analysis of two clinical trials revealed no differences between PsO patients with and without MetS (84). Nevertheless, no evidence on its impact on MetS components in PsA is available. Due to the functional differences in the IL-23/IL-17 axis across chronic inflammatory conditions (85, 86), and the role of IL-23 in maintaining gut homeostasis and preventing obesity in animal models (87), studies on the effect of IL-23 blockade in metabolic outcomes in PsA patients are warranted.

### Small Molecules

#### Apremilast

Apremilast is a phosphodiesterase 4 (PDE4) inhibitor belonging to the class of oral small molecules. It is indicated for the treatment of PsA and moderate/severe PsO (88). It acts at the intracellular level by modulating the production of pro-inflammatory and anti-inflammatory mediators by PDE4. In addition to fueling inflammatory processes, PDE4 seems to be also involved in lipid and glucose metabolism disorders, liver steatosis, altered lipolysis, and neuroendocrine alterations (89, 90). Therefore, its inhibition may bring benefits on both the inflammatory component at the base of PsO/PsA, as well as on the MetS components.

Inhibition of PDE4 improves liver steatosis, reduces lipid deposition in the liver and consequently improves insulin resistance (89). Apremilast also appears to contribute to counteracting endothelial dysfunction, thus reducing CV risk (91, 92), and to stabilize atherosclerotic plaques, hence blocking its evolution to an unstable, high risk phenotype (93). An interesting study conducted by Mazzilli and coworkers observed a better therapeutic response to apremilast in diabetic patients compared to non-diabetic patients, with a reduction in blood glucose and total- and LDLc levels (94). Based on these findings, apremilast may be an appropriate therapeutic choice in patients with PsO/PsA and MetS (94).

#### Tofacitinib

Tofacitinib is an oral Janus kinase inhibitor (JAKi) that works interfering with the intracellular signaling pathway of s number of cytokines and inflammatory mediators. It is indicated for the treatment of PsA (95–97). Tofacitinib treatment has been observed to increase LDLc levels (98). Hypercholesterolemia is an important CV risk factor, and for this reason tests have been carried out aimed at assessing the efficacy and safety of tofacitinib in patients with MetS, and in general in those with increased CV risk (98, 99). A *post-hoc* analysis of phase III tofacitinib studies analyzed the efficacy and safety profile of this drug in patients with MetS (99).

Regarding efficacy data, the proportion of patients with MetS reaching endpoints such as ACR20/50/70 and Health Assessment Questionnaire-Disability Index (HAQ-DI) response, as well as resolution of enthesitis and dactilytis, was greater in the tofacitinib group compare to placebo. When comparing patients with and without MetS, similar results were observed except for resolution of dactylitis and HAQ-DI response, which were lower in patients with MetS. Regarding safety data, no differences in the proportion of adverse events were found between tofacitinib and placebo groups, and no new risk factors were identified in tofacitinib-treated presenting MetS at baseline (99). Since patients with MetS are much more likely to develop non-alcoholic fatty liver disease (100), and considering the tofacitinib-induced hyperlipidemia, the hepatic impact of tofacitinib in this subset of patients was analyzed, and no clinically relevant abnormalities were found (99).

Considering the increased CV risk in PsA patients, Dafna and coworkers analyzed changes in lipid profile, risk factors for CV disease occurrence, and incidence of MACE in patients with PsA undergoing treatment with tofacitinib (5 or 10 mg twice a day) in combination with conventional DMARDs (98). Although a 10–15% increase in lipid levels was observed, HDLc was increased in conjunction with other lipids, and no significant changes were observed in the LDLc/HDLc or total cholesterol/HDLc ratios (98). A parallel, significant reduction in CRP levels was also registered (98). Importantly, lipid ratios and CRP levels and blood pressure/hypertension are known CV risk factors in PsA (101–103). Therefore, these findings did not show overall a further increased risk of CV disease after treatment with tofacitinib (98), thus suggesting that these lipid changes do not fully translate into CV disease occurrence. Similar picture has been also observed in RA (32, 104).

## Unmet Needs, Future Perspectives and Conclusions

Compelling evidence urges the intervention of cardiometabolic risk in PsA patients. Due to the involvement of inflammatory pathways on MetS components, the use of DMARDs may be accompanied by a MetS mitigation. This aligns with the EULAR recommendations of keeping a tight disease control and flare prevention in order to achieve a good CV risk management (105). Overall, the current literature is supportive of a therapeutic effect of approved DMARDs on MetS outcomes in PsA populations. However, the whole picture is far from being clear and the existing evidence is not optimal for a robust therapeutic decision making process in PsA. Of note, DMARDs may be double-edge swords in this scenario. For example, corticosteroids worsen glucose homeostasis, and NSAIDs are associated with an increased CV risk (4). On the contrary, other treatments such as TNFi may decrease the cardiometabolic risk by reducing the underlying inflammatory response in PsA (63, 106). Therefore, the MetS burden may be at the center of the therapeutic decision process, in addition to joint and skin involvement. This is in line with the most recent international recommendations for PsA management, which highlight the relevance of comorbidities to choose the most appropriate drug for each patient and tailor therapeutic approaches in accordance (107–109).

Unfortunately, the evidence on the harmful/beneficial balance effect of the different DMARDs approved for PsA is far from being optimal, and there is a lack of robust evidence to guide these decisions in PsA populations. On the one hand, most of the evidence in terms of CV effects derive from RA and PsO studies. Although similarities between RA and PsA exist (59), significant differences in terms of pathogenesis are present, especially regarding CD4^+^ T-cell involvement, TNF/IL-17 role and participation of the humoral response. More importantly, traditional CV risk factors are overrepresented in PsA compared to RA populations, although CV disease occurrence seems to be higher in the latter, hence pointing to divergent patterns in the inflammation-cardiometabolic risk connection across diseases. Of note, the levels of evidence of the recommendations for CV risk management in PsA is lower than those of RA (105). Taken together, all these lines of evidence emphasize the need for further, well-conducted PsA-specific studies. In fact, in a complex clinical scenario as PsA, a special attention should be paid to the comorbidity-multimorbidity spectrum (3). Multimorbidity could be a novel driving force in improving the disease management by giving a role for the several conditions potentially coexisting in PsA, shifting from a classical “index disease” model to a “multimorbidity centered” scheme. The role of patient preferences and patient-centric concepts is warranted in this setting.

In addition to better studies and comparative trials with the drugs therein reviewed, there is a clear knowledge gap in terms of the clinical effects on the MetS and its components of the approved drugs targeting the IL-17/IL-23 axis in PsA patients. Individual and head-to-head comparative clinical trials are much awaited due to the clinical benefit of these drugs in joint and skin domains in PsA patients. However, to which extent this clinical benefit translates to MetS, CV or multimorbidity outcomes in PsA patients remain unclear. Similarly, whether these drugs may benefit the general PsA population or specific patient subsets represent an important unmet need in this setting. Additionally, it is important to note that the existing evidence came from pharmaco-epidemiological studies, and thus are inherently affected by allocation and confounding by indication biases, which are a key limitation to establish firm recommendations for clinical practice. The need for better design trials and large registries to address this questions is in the research agenda (104).

Besides the effect of DMARDs on MetS outcomes, PsA patients face important rates of underscreening, underdiagnosis, and undertreatment of CV risk factors, including MetS components [reviewed in (59)]. This poses relevant questions in terms of cardiometabolic risk intervention. First, it may be important to ascertain whether the documented effect of DMARDs in the existing studies may be an underestimation of their actual effect due to poor risk factor conventional treatment (anti-hypertensive, lipid-lowering, oral antidiabetic agents, etc.). Second, it may be key to elucidate if DMARDs plus conventional risk factor conventional treatments show potential synergistic effects or if drug-drug interactions should be considered. Third, whether an optimal management of CV risk factors leads to a better disease control by virtue of the bi-directional crosstalk between cardiometabolic and inflammatory pathways remain to be elucidated.

Finally, the role of lifestyle interventions should be considered in future studies and clinical research. EULAR urges the implementation of lifestyle modifications to dampen CV risk factors. Whether these interventions can add to or modulate the effect of DMARDs on the MetS burden needs to be established. With the advancement of the syndemics framework for complex conditions (110, 111), such an approach must be conceived in this scenario.

## Author Contributions

FA, EG, IF, SB, and JR-C designed and performed the literature search. FA and JR-C drafted the narrative review and edited the manuscript. All authors read the manuscript, revised it for intellectual content, approved the final version, and agree to be accountable for all aspects of the work.

## Conflict of Interest

The authors declare that the research was conducted in the absence of any commercial or financial relationships that could be construed as a potential conflict of interest.

## Publisher's Note

All claims expressed in this article are solely those of the authors and do not necessarily represent those of their affiliated organizations, or those of the publisher, the editors and the reviewers. Any product that may be evaluated in this article, or claim that may be made by its manufacturer, is not guaranteed or endorsed by the publisher.
